# Subcutaneous allergen immunotherapy with dermatophagoides pteronyssinus in patient with local allergic rhinitis

**DOI:** 10.1186/1939-4551-8-S1-A263

**Published:** 2015-04-08

**Authors:** Carmen Rondon, Paloma Campo, Natalia Blanca-López, Francisca Gomez, Maria Dolores Ruiz, Gabriela Canto, Maria Jose Torres, Miguel Blanca

**Affiliations:** 1 Allergy Unit, Regional University Hospital of Málaga, Spain; 2 Allergy Service, Hospital Infanta Leonor, Spain

## Background

In this study we investigated the efficacy and safety of subcutaneous allergen immunotherapy (AIT) with *Dermatophagoides pteronyssinus* (DP) in patients with local allergic rhinitis (LAR).

## Methods

A randomized, double-blind, placebo-controled, parallel-group, phase II study was conducted. Thirty-six subjects with LAR to DP were randomized to receive Pangraminâ PLUS, ALK, *Dermatophagoides pteronyssinus* (AIT-DP) or placebo for 24 months. The primary endpoint was total symptoms (TSS) and total medication scores (TMS). Secondary endpoints included: total combined symptom+medication scores (TCS), daily symptoms score (DSS), daily medication score (DMS), medication free days (MFD), skin testing, nasal allergen provocation test (NAPT-DP), and adverse events. Serum and nasal lavage samples were obtained for immunological studies.

## Results

Twenty-eight patients completed the study. AIT-DP produced a significant improvement in the primary endpoints compared to placebo (a 47% of reduction in TSS (0.60 vs 1.14; p<0.001) and a 51.2% in TMS (0.65 vs 1.34; p=0.002). Moreover, at 6-12-18-24 months significant improvements in TCS (p=0.046; p=0.037; p=0.011; p=0.007) and DSS (p=0.003; p=0.012; p<0.001; p<0.001); and at 24 months in DMS (p=0.014), and MFD (p=0.031) compared to placebo were observed. AIT-DP induced an objective improvement in nasal tolerance to NAPT-DP at 6-12-18-24 months (p=0.003; p<0.001; p<0.001; p<0.001) compared to placebo, with negative responses in the 50% of patients. AIT-DP was well-tolerated, one patient had a local moderate reaction solved without systemic treatment. No systemic reactions occurred.

## Conclusion

We prove that AIT with *Dermatophagoides Pteronyssinus* is an effective and well-tolerated treatment in LAR patients. This phase II study provides the indication for AIT in LAR.

